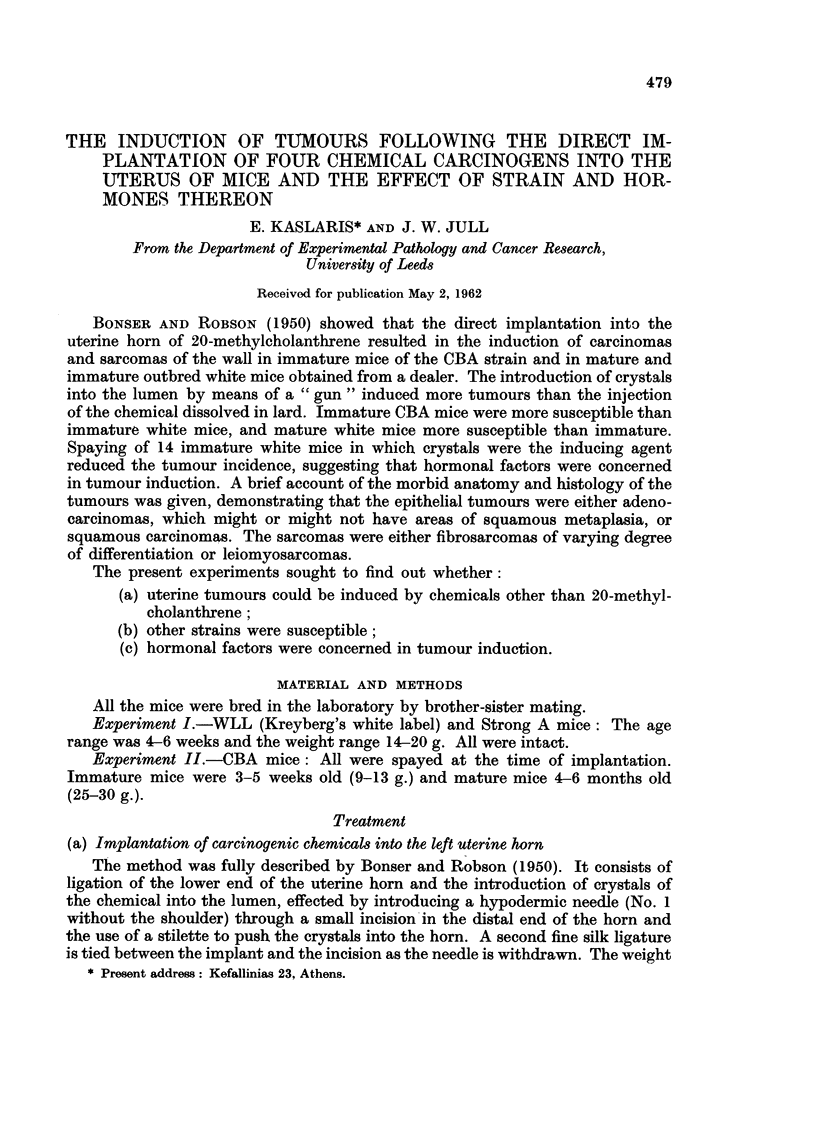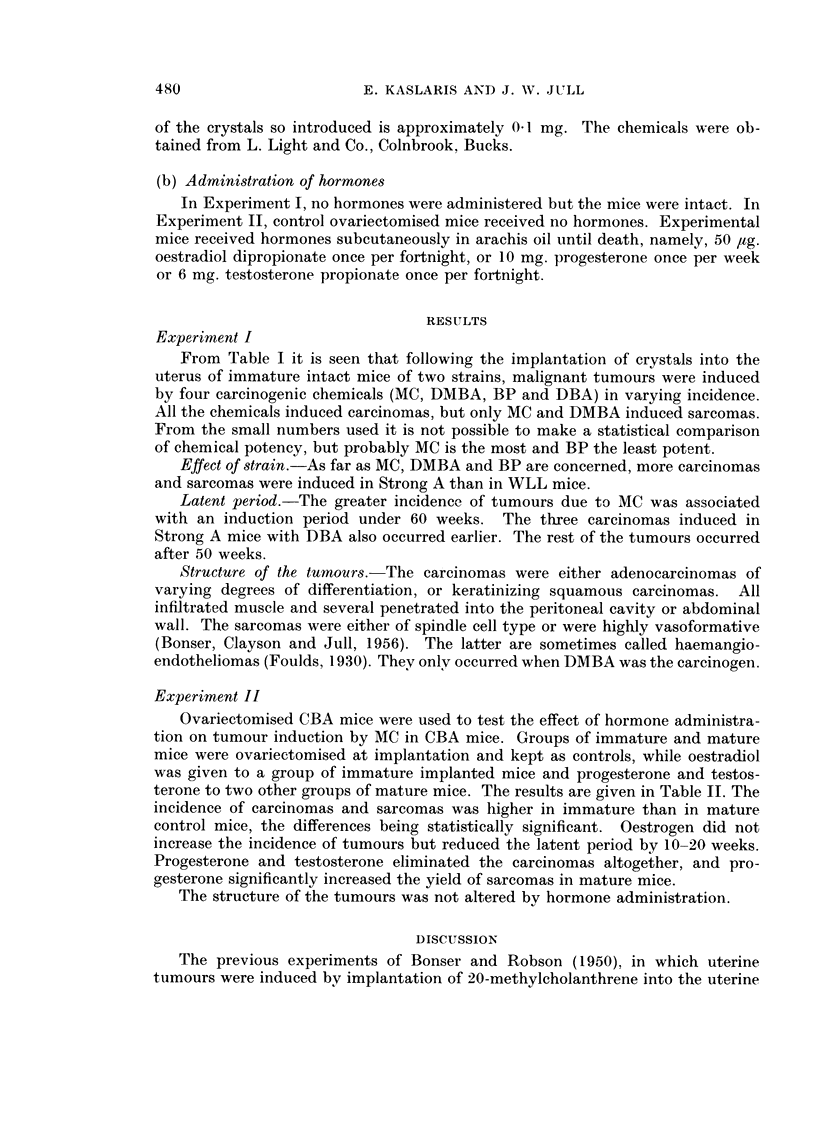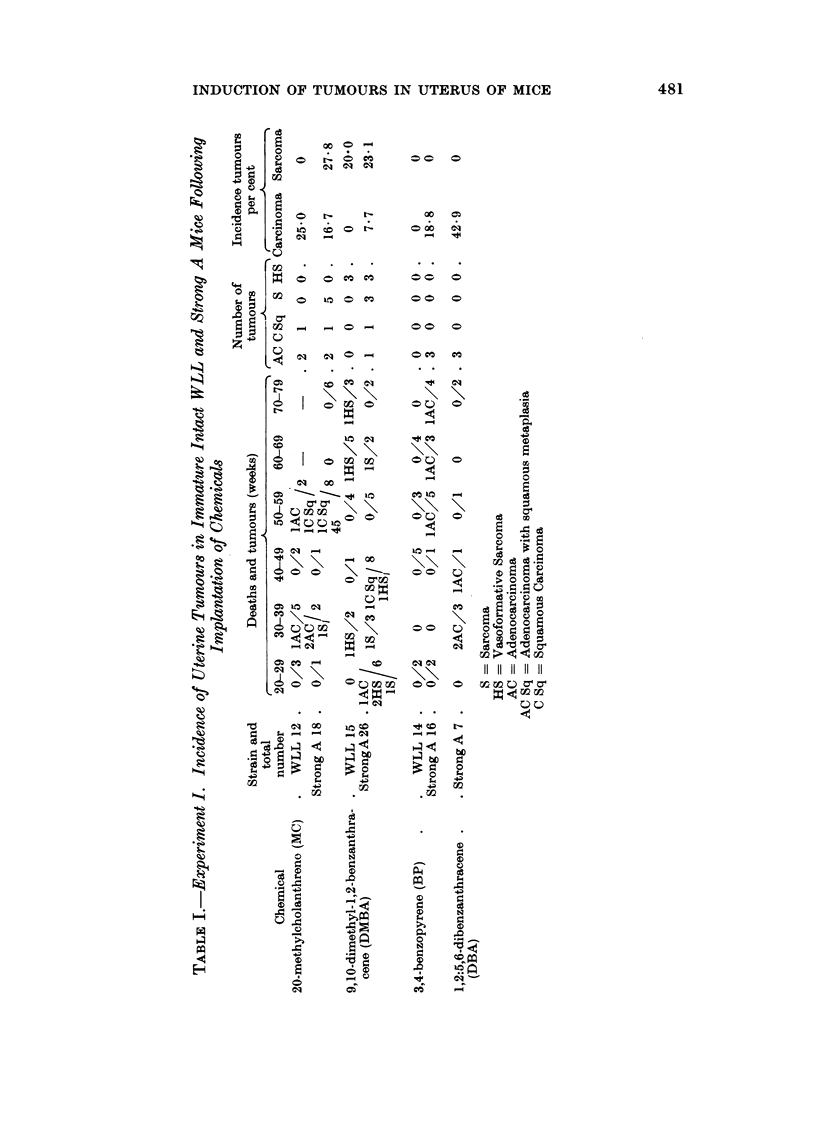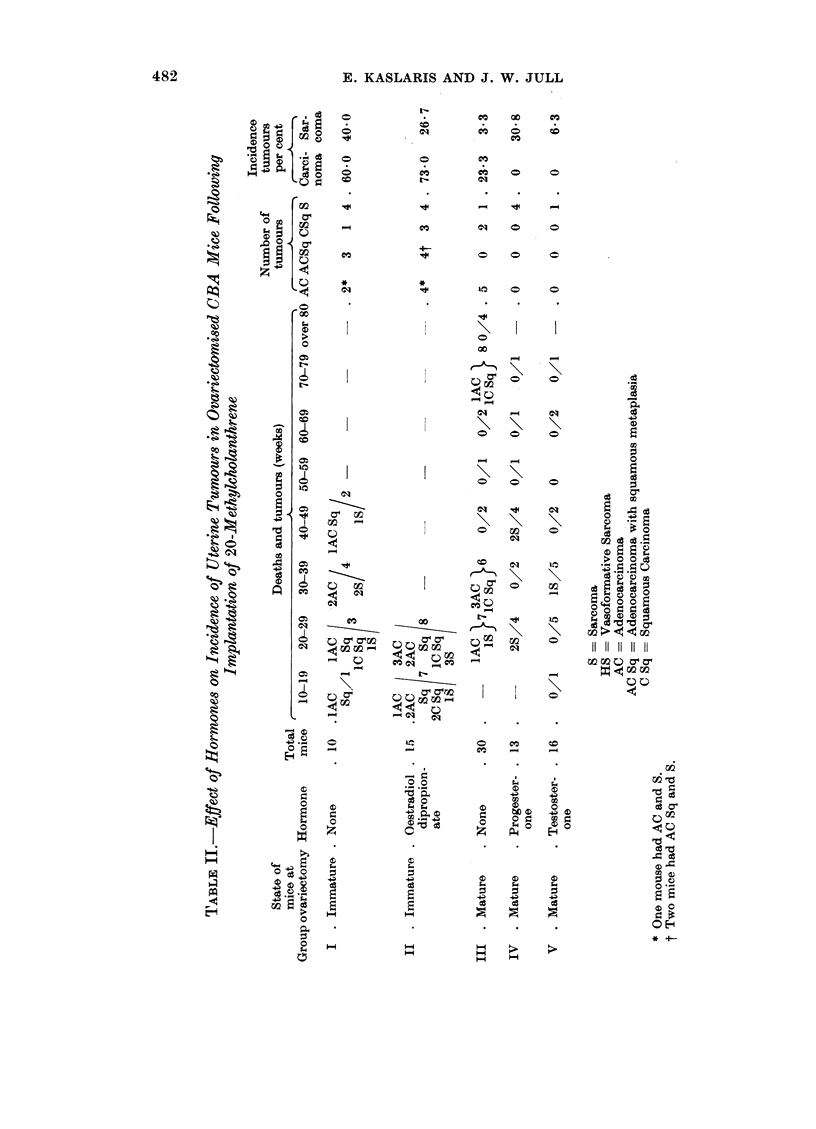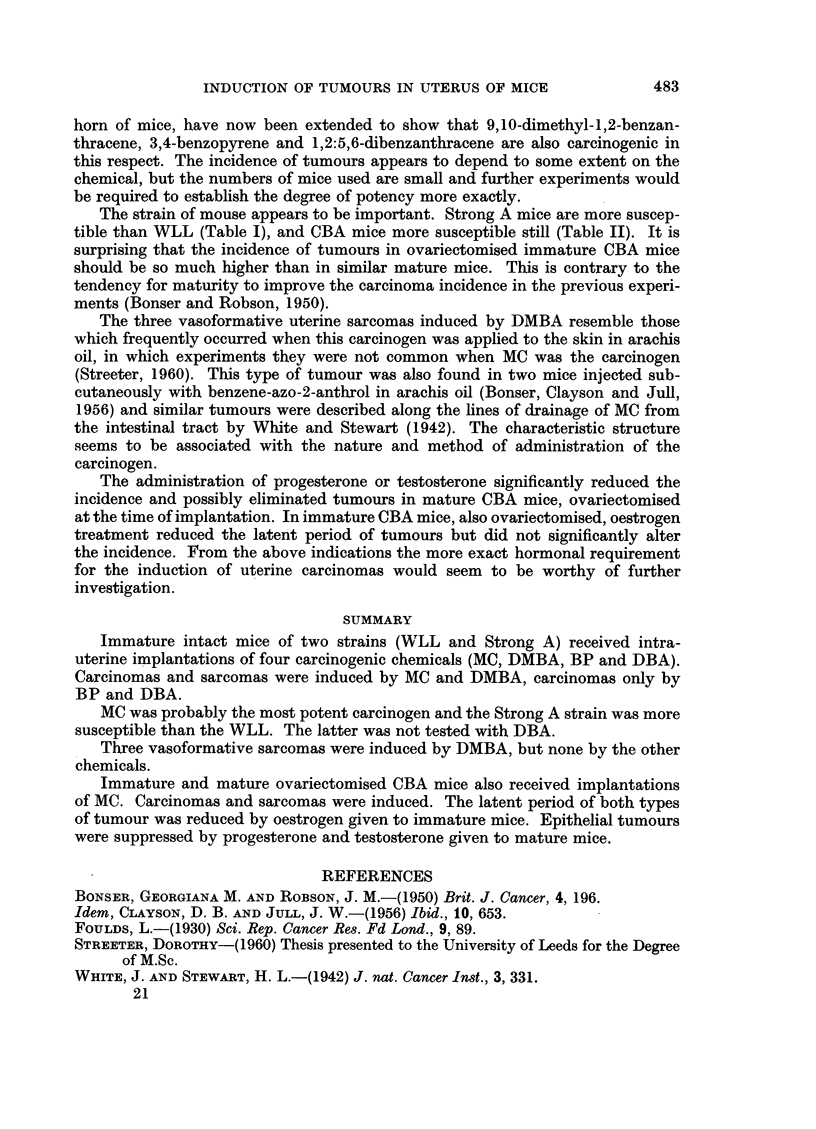# The Induction of Tumours following the Direct Implantation of Four Chemical Carcinogens into the Uterus of Mice and the Effect of Strain and Hormones Thereon

**DOI:** 10.1038/bjc.1962.54

**Published:** 1962-09

**Authors:** E. Kaslaris, J. W. Jull


### THE INDUCTION OF TUMOURS FOLLOWING THE DIRECT IMPLANTATION OF FOUR CHEMICAL CARCINOGENS INTO THE UTERUS OF MICE AND THE EFFECT OF STRAIN AND HORMONES THEREON

E. KASLARIS* AND J. W. JULL

*From the Department of Experimental Pathology and Cancer Research, University of Leeds*

Received for publication May 2, 1962

BONSER AND ROBSON (1950) showed that the direct implantation into the uterine horn of 20-methylcholanthrene resulted in the induction of carcinomas and sarcomas of the wall in immature mice of the CBA strain and in mature and immature outbred white mice obtained from a dealer. The introduction of crystals into the lumen by means of a " gun " induced more tumours than the injection of the chemical dissolved in lard. Immature CBA mice were more susceptible than immature white mice, and mature white mice more susceptible than immature. Spaying of 14 immature white mice in which crystals were the inducing agent reduced the tumour incidence, suggesting that hormonal factors were concerned in tumour induction. A brief account of the morbid anatomy and histology of the tumours was given, demonstrating that the epithelial tumours were either adenocarcinomas, which might or might not have areas of squamous metaplasia, or squamous carcinomas. The sarcomas were either fibrosarcomas of varying degree of differentiation or leiomyosarcomas.

The present experiments sought to find out whether :

(a) uterine tumours could be induced by chemicals other than 20-methylcholanthrene ;
(b) other strains were susceptible ;
(c) hormonal factors were concerned in tumour induction.

### MATERIAL AND METHODS

All the mice were bred in the laboratory by brother-sister mating.

*Experiment I.*—WLL (Kreyberg's white label) and Strong A mice : The age range was 4–6 weeks and the weight range 14–20 g. All were intact.

*Experiment II.*—CBA mice : All were spayed at the time of implantation. Immature mice were 3–5 weeks old (9–13 g.) and mature mice 4–6 months old (25–30 g.).

### *Treatment*

#### (a) *Implantation of carcinogenic chemicals into the left uterine horn*

The method was fully described by Bonser and Robson (1950). It consists of ligation of the lower end of the uterine horn and the introduction of crystals of the chemical into the lumen, effected by introducing a hypodermic needle (No. 1 without the shoulder) through a small incision in the distal end of the horn and the use of a stilette to push the crystals into the horn. A second fine silk ligature is tied between the implant and the incision as the needle is withdrawn. The weight

* Present address : Kefallinias 23, Athens.

of the crystals so introduced is approximately 0·1 mg. The chemicals were obtained from L. Light and Co., Colnbrook, Bucks.

(b) *Administration of hormones*

In Experiment I, no hormones were administered but the mice were intact. In Experiment II, control ovariectomised mice received no hormones. Experimental mice received hormones subcutaneously in arachis oil until death, namely, 50 $\mu$g. oestradiol dipropionate once per fortnight, or 10 mg. progesterone once per week or 6 mg. testosterone propionate once per fortnight.

### RESULTS

*Experiment I*

From Table I it is seen that following the implantation of crystals into the uterus of immature intact mice of two strains, malignant tumours were induced by four carcinogenic chemicals (MC, DMBA, BP and DBA) in varying incidence. All the chemicals induced carcinomas, but only MC and DMBA induced sarcomas. From the small numbers used it is not possible to make a statistical comparison of chemical potency, but probably MC is the most and BP the least potent.

*Effect of strain.*—As far as MC, DMBA and BP are concerned, more carcinomas and sarcomas were induced in Strong A than in WLL mice.

*Latent period.*—The greater incidence of tumours due to MC was associated with an induction period under 60 weeks. The three carcinomas induced in Strong A mice with DBA also occurred earlier. The rest of the tumours occurred after 50 weeks.

*Structure of the tumours.*—The carcinomas were either adenocarcinomas of varying degrees of differentiation, or keratinizing squamous carcinomas. All infiltrated muscle and several penetrated into the peritoneal cavity or abdominal wall. The sarcomas were either of spindle cell type or were highly vasoformative (Bonser, Clayson and Jull, 1956). The latter are sometimes called haemangio-endotheliomas (Foulds, 1930). They only occurred when DMBA was the carcinogen.

*Experiment II*

Ovariectomised CBA mice were used to test the effect of hormone administration on tumour induction by MC in CBA mice. Groups of immature and mature mice were ovariectomised at implantation and kept as controls, while oestradiol was given to a group of immature implanted mice and progesterone and testosterone to two other groups of mature mice. The results are given in Table II. The incidence of carcinomas and sarcomas was higher in immature than in mature control mice, the differences being statistically significant. Oestrogen did not increase the incidence of tumours but reduced the latent period by 10–20 weeks. Progesterone and testosterone eliminated the carcinomas altogether, and progesterone significantly increased the yield of sarcomas in mature mice.

The structure of the tumours was not altered by hormone administration.

### DISCUSSION

The previous experiments of Bonser and Robson (1950), in which uterine tumours were induced by implantation of 20-methylcholanthrene into the uterine horn of mice, have now been extended to show that 9,10-dimethyl-1,2-benzanthracene, 3,4-benzopyrene and 1,2:5,6-dibenzanthracene are also carcinogenic in this respect. The incidence of tumours appears to depend to some extent on the chemical, but the numbers of mice used are small and further experiments would be required to establish the degree of potency more exactly.

The strain of mouse appears to be important. Strong A mice are more susceptible than WLL (Table I), and CBA mice more susceptible still (Table II). It is surprising that the incidence of tumours in ovariectomised immature CBA mice should be so much higher than in similar mature mice. This is contrary to the tendency for maturity to improve the carcinoma incidence in the previous experiments (Bonser and Robson, 1950).

The three vasoformative uterine sarcomas induced by DMBA resemble those which frequently occurred when this carcinogen was applied to the skin in arachis oil, in which experiments they were not common when MC was the carcinogen (Streeter, 1960). This type of tumour was also found in two mice injected subcutaneously with benzene-azo-2-anthrol in arachis oil (Bonser, Clayson and Jull, 1956) and similar tumours were described along the lines of drainage of MC from the intestinal tract by White and Stewart (1942). The characteristic structure seems to be associated with the nature and method of administration of the carcinogen.

The administration of progesterone or testosterone significantly reduced the incidence and possibly eliminated tumours in mature CBA mice, ovariectomised at the time of implantation. In immature CBA mice, also ovariectomised, oestrogen treatment reduced the latent period of tumours but did not significantly alter the incidence. From the above indications the more exact hormonal requirement for the induction of uterine carcinomas would seem to be worthy of further investigation.

### SUMMARY

Immature intact mice of two strains (WLL and Strong A) received intra-uterine implantations of four carcinogenic chemicals (MC, DMBA, BP and DBA). Carcinomas and sarcomas were induced by MC and DMBA, carcinomas only by BP and DBA.

MC was probably the most potent carcinogen and the Strong A strain was more susceptible than the WLL. The latter was not tested with DBA.

Three vasoformative sarcomas were induced by DMBA, but none by the other chemicals.

Immature and mature ovariectomised CBA mice also received implantations of MC. Carcinomas and sarcomas were induced. The latent period of both types of tumour was reduced by oestrogen given to immature mice. Epithelial tumours were suppressed by progesterone and testosterone given to mature mice.

### REFERENCES

Bonser, Georgiana M. and Robson, J. M.—(1950) *Brit. J. Cancer*, **4**, 196.
*Idem*, Clayson, D. B. and Jull, J. W.—(1956) *Ibid.*, **10**, 653.
Foulds, L.—(1930) *Sci. Rep. Cancer Res. Fd Lond.*, **9**, 89.
Streeter, Dorothy—(1960) Thesis presented to the University of Leeds for the Degree of M.Sc.
White, J. and Stewart, H. L.—(1942) *J. nat. Cancer Inst.*, **3**, 331.